# Domestic

**Published:** 1842-02-12

**Authors:** 


### DOMESTIC.

*Note in reply to the letter of Dr. Ticknor, contained in the last number of the Examiner.* By W. S. W. Ruschenberger, M. D.—I regret to perceive that Dr. Ticknor is aggrieved by the report of a case of "Deformed Leg," in the second number of your journal.

Dr. Ticknor asserts in his letter, published in the sixth number of the Medical Examiner, that "the *only* deformity existing in the case of Lieutenant M———" was "a shortening of half an inch." The description of the leg prior to the operation, given by me, the accompanying woodcut taken from a plaster cast of the leg, and the cast itself, furnish sufficient evidence, as respects the nature and degree of deformity that existed; and, surely, no one can for a moment suppose that Lieutenant M——— would submit to a severe and dangerous operation—that a distinguished surgeon in a Christian community would perform such an operation—or that respectable surgeons would permit or advise such an operation, without having a prospect of some benefit to be derived to the patient. I feel sure that Dr. Ticknor does not think that such operations are performed merely for the sake of operating, although such might be inferred from his words—"the only deformity," &c.

Dr. Ticknor finds fault with the whole title of the report—and particularly with the words "unsuccessfully treated," which he does not think applicable.

As Dr. T. argues that the words "unsuccessfully treated" implies a mismanagement of the case, it may be well to bear in mind that want of success, that is, a happy result, may be the consequence of the most judicious treatment, under the circumstances; and, consequently, a surgeon might even merit praise for succeeding in any degree approaching towards a perfect cure.

[We have inserted the above note of Dr. Ruschenberger as much in justice to the operator as the reporter of the case. The performance of so serious an operation for the mere purpose of removing a deformity consisting of a slight shortening of the limb would be pronounced wholly unwarrantable, and the excision of bone would strongly tend to increase instead of diminishing the shortening. The operation was performed for the removal of the angular displacement, and that observed in the direction of the diameter of the bones, and not to prevent the simple overlapping of the fragments of a very oblique fracture. The plaster cast of the limb, which cannot possibly exaggerate, plainly tells the character of the deformity, and the wood cut presents an unusually faithful copy of the cast.

But, assuredly, it would be wrong for our readers, or ourselves, to attribute the unsuccessful result of the original treatment to any fault in the surgeon, without further knowledge of the apparatus employed, and the various incidental circumstances surrounding it, than has yet been given to the public. The difficulties attending the treatment of fractures at sea, would furnish an excellent subject for an article of some length.] R. C.

